# Azole-Resistant *Aspergillus fumigatus*: Epidemiology, Diagnosis, and Treatment Considerations

**DOI:** 10.3390/jof11100731

**Published:** 2025-10-10

**Authors:** Anna Zubovskaia

**Affiliations:** Division of Infectious Diseases, Wellstar MCG, Augusta University, Augusta, GA 30912, USA; azubovskaia@augusta.edu

**Keywords:** *Aspergillus fumigatus*, azole resistance, *cyp51A*, antifungal resistance, invasive aspergillosis

## Abstract

Invasive aspergillosis is an opportunistic infection caused by the *Aspergillus* species. It is a significant cause of morbidity and mortality in susceptible populations, including recipients of bone marrow and solid organ transplants. Azole antifungals have remained the first-line treatment for invasive aspergillosis for a long time; however, the advance of azole resistance in *Aspergillus fumigatus*, driven predominantly by extensive commercial and agricultural use of azole fungicides and environmental exposure of susceptible populations to the resistant strains, renders the traditional therapeutic approaches less effective and results in further increase in mortality. The epidemiology, molecular mechanisms of azole resistance, diagnostic approaches, and clinical implications of azole resistance in *Aspergillus fumigatus sensu stricto* will be discussed in this article (for ease of comprehension, the rest of this article will refer to *A. fumigatus sensu stricto* as *A. fumigatus*).

## 1. Introduction

*A. fumigatus* is a hyaline mold, which is the most common cause of invasive aspergillosis worldwide [1]. *Aspergillus* rarely causes infections in patients with a normally functioning immune system; however, it can cause life-threatening disease in immunocompromised patients, including patients with hematological malignancies, solid organ transplants, and recipients of allo-stem cell transplants [2,3]. Additionally, infections caused by *A. fumigatus* may complicate viral pneumonias, such as influenza and COVID-19, increasing the mortality among patients with these diseases, including patients in the intensive care units [4]. Infections due to *Aspergillus* are a cause of significant morbidity and mortality worldwide. According to a study by Benedict et al., hospitalizations for *Aspergillus* infections also cause a significant financial impact, accounting (along with *Candida*) for the most hospitalizations among the fungal infections and the highest total hospitalization cost among the fungal diseases [5]. The global burden of allergic bronchopulmonary aspergillosis has been estimated to exceed 4.8 million people worldwide, with an estimated 2.5% prevalence among adults with asthma in one study [6]. Chronic pulmonary aspergillosis often complicates other pulmonary diseases, such as tuberculosis, sarcoidosis, and chronic obstructive pulmonary disease, etc., as well as cases of allergic bronchopulmonary aspergillosis, and causes significant morbidity worldwide [6,7].

The development of azole-resistance in *A. fumigatus* occurs via two mechanisms: 1—during prolonged exposure of a human host to azole therapy and 2—due to azole use as fungicides, leading to *A. fumigatus* exposure to fungicidal agents in the environment and development of cross-resistance to medical azoles [1,8,9,10,11,12]. Studies have established that the selective pressure from the triazole fungicides in the environment leads to the development of cross-resistance to medical azoles and is currently considered the main driver of the development of azole resistance [11]. Extensive utilization of azoles in agriculture exerts selection pressure on environmental *Aspergillus* strains, contributing to the emergence of resistant strains in the environment, as well as during human infection [13].

The studies suggest that azole resistance leads to higher mortality and worse outcomes in patients with azole-resistant invasive aspergillosis [14]. The comparison of mortality between patients with voriconazole-susceptible invasive *A. fumigatus* infection and voriconazole-resistant infection revealed an excess overall mortality of 25% at day 90 [15]. Diagnosis and management of azole-resistant aspergillosis pose a significant challenge. In this article, we review the current literature evidence on epidemiology, diagnosis, and management of azole-resistant *A. fumigatus sensu stricto* (throughout the article we will use the term *A. fumigatus* for *A. fumigatus sensu stricto*).

## 2. Epidemiology of Azole-Resistant *A. fumigatus*

Although cases of azole-resistant *A. fumigatus* have been reported in many regions worldwide, data on the incidence and prevalence of azole-resistant *A. fumigatus* vary.

Studies from Europe show significant regional variation in the prevalence of infections due to azole-resistant *A. fumigatus* [1]. The data utilized in studies and reports come from both environmental surveillance studies and surveillance of isolates from patients with clinical diseases caused by *A. fumigatus*, making direct comparison of the data challenging. As part of the SENTRY Antifungal Surveillance Program 449, *A. fumigatus* isolates from Europe from patients with invasive aspergillosis were tested for azole resistance. The susceptibility was determined by CLSI (Clinical & Laboratory Standards Institute) M38 broth microdilution testing utilizing epidemiological cutoff values (ECV), and an ECV of 0.5 mg/L for posaconazole was utilized. Among the tested isolates, 10.7% were non-wild type to one or more azoles, with 7.3% being non-wild type to isavuconazole, 9.1% to itraconazole, 3.6% to posaconazole, and 4.5% to voriconazole [16]. In the recently published study by Song et al., clinical *A. fumigatus* isolates from Dutch hospitals collected from 6 January 1994, until 31 December 2022, were evaluated for azole resistance (*A. fumigatus* confirmed by mycological methods and β-tubulin sequencing). Susceptibility testing was performed using the EUCAST (European Committee on Antimicrobial Susceptibility Testing) microdilution reference method, and the isolates with triazole-resistant phenotypes were further analyzed for the presence of resistance-conferring mutations by PCR amplification and sequencing. This study showed that 15.6% (1979/12679) of isolates harbored resistance-associated mutations, with 67.6% harboring TR34/Leu98His *sensu stricto* mutation and TR46/Tyr121Phe/Thr289Ala sensu stricto detected in 16.8% of isolates [17]. Pan-azole resistance was detected among 916 (68.5%) of the TR34/Leu98His *sensu stricto* mutants and 106 (31.9%) of the TR46/Tyr121Phe/Thr289Ala *sensu stricto* isolates, with a total of 1920 (97%) of all triazole-resistant isolates being voriconazole resistant [18]. Azole-resistant strains were detected among 13/59 cases of proven or probable invasive aspergillosis, with 11/13 (84.6%) caused by mixed genotype infection [17]. The one-year prospective surveillance at the French cystic fibrosis reference center evaluated the prevalence of azole resistance among screened isolates of *Aspergillus* section *Fumigati* from colonized patients. *A. fumigatus* strains were identified by partial β-tubulin sequencing, and azole resistance screening was performed by in-house protocol derived from the EUCAST broth microdilution, and the MICs of azoles were interpreted using the EUCAST breakpoints (version 8.1). Of all tested isolates, 6.5% were resistant to at least one azole, with the most frequently isolated mutation being TR34/L98H [18].

The susceptibility trends towards five antifungal agents among *A. fumigatus* isolates obtained over 12 years at the University Hospital Essen, Germany, were assessed by the broth microdilution method following utilizing EUCAST recommendations, and the presence of the most common mutations was determined by utilizing multiplex real-time PCR (AsperGenius^®^ 1.0, Pathonostics, Maastricht, the Netherlands) [19]. Among 143 clinical isolates of azole-resistant *A. fumigatus* (identified by mycological methods and by β-tubulin or ITS sequencing), 97.9% were resistant to voriconazole, and 92.3% were resistant to itraconazole. Out of 143 tested azole-resistant isolates, 123 (86%) harbored the TR34/L98H mutation, whereas TR46/Y121F/T289A was detected in 7% of isolates, and other *cyp51A* mutations were responsible for azole resistance in an additional 7% of isolates [19]. The TR46/Y121F/T289A and other *cyp51A* mutations were progressively more often identified after 2016, whereas before 2016 the TR34/L98H was the most identified underlying resistance mutation [19].

The Danish National Surveillance study from 2018 to 2020 to evaluate the prevalence of azole resistance among *A. fumigatus isolates* [20]. Only *Aspergillus fumigatus sensu stricto* isolates were included in the study (identified by mycological methods, thermotolerance testing with MALDI-TOF, and β-tubulin sequencing), and susceptibility testing was performed using the EUCAST method with EUCAST susceptibility breakpoints (version 10.0). The *cyp51A* sequencing was performed for isolates resistant to at least one azole. The prevalence of resistance to at least one azole was estimated at 6.1%, with itraconazole resistance among 5.9% of isolates and voriconazole resistance among 5.6% of isolates [20]. The TR34/L98H mutations were present in 3.6% (59.1% of resistant isolates). The other *cyp51A* mutations and non-*cyp51A*-mediated resistance mechanisms were detected in 21.2% and 19.7% of azole-resistant isolates, respectively [20]. In the study by Escribano et al., the isolates from 30 hospitals in Spain were identified using MALDI-TOF and tested for susceptibility utilizing the EUCAST breakpoints, with *A. fumigatus sensu stricto* resistant isolates undergoing sequencing of the *cyp51A* gene [21]. Among the *A. fumigatus sensu stricto* isolates, 45/828 (5.5%) were resistant to at least one azole, with the highest rate of resistance to voriconazole [21]. According to the recently published prospective surveillance of 335 clinical and environmental isolates collected over a 3-year period from a single Spanish hospital (identification to the species level and susceptibility testing using the EUCAST broth microdilution reference method), only 2 azole-resistant *A. fumigatus* strains were detected [22].

The routine testing of the *A. fumigatus* isolates susceptibility to azoles in the US is not recommended by the IDSA guidance and is not considered a standard of care, so the true prevalence is unknown [23]. The studies suggest that the environmentally occurring resistance to azoles, mediated through *cyp51A* mutations, including TR34/L98H (TR34) and TR46/Y121F/T289A (TR46), is becoming an emerging problem in the US. The passive surveillance conducted in the US in 2011–2013 on 1026 clinical isolates of *A. fumigatus* from 22 states detected no TR34/L98H or TR46/Y121F/T289A mutations in any of the tested isolates. About 5% of isolates exhibited increased MICs to itraconazole by screening on antifungal plate culture and itraconazole Etest [24]. The I242V mutation was the most commonly identified among isolates with higher MICs to itraconazole, and only the M220I mutation was associated with treatment failure [24]. In the passive surveillance conducted by Berkow et al. in 2015–2017, 1.4% of isolates exhibited elevated MICs to one or both tested azoles (itraconazole and voriconazole). The species confirmation was conducted, as previously described in the study by Pham et al., and the isolates with elevated MICs detected on Etest were subsequently tested by the broth microdilution test per CLSI guidelines. The ECVs (epidemiologic cutoff values) were utilized for susceptibility determination [24,25]. Among the resistant isolates, 14/20 contained an amino acid substitution in *cyp51*, and the TR34/L98H mutation was detected in 5 isolates [25]. Due to the voluntary submission of the isolates for surveillance, there was a nonuniform geographical distribution of samples, and the authors suggested that these data substantially underestimate the true prevalence of the azole-resistant *A. fumigatus* in the US [23]. The foundations of azole resistance were evaluated on 179 clinical and environmental *A. fumigatus* isolates from the US and 18 non-US genomes, which suggested a common origin of the TR34/L98H among internationally sourced and US isolates, whereas mainly US-sourced isolates demonstrated an absence of the TR34/L98H mutation [26]. In this study, 26% of the US-sourced isolates showed resistance to azoles (either voriconazole or itraconazole) [26]. The study of environmentally sourced isolates suggests that azole-resistant *A. fumigatus* may be more widespread in the US than previously thought [27]. The retail plant products in the Georgia area were evaluated for resistance to the fungicide tebuconazole, voriconazole, itraconazole, and posaconazole, using the EUCAST breakpoints [28]. Isolates resistant to more than 1 medical azole or 1 medical azole plus tebuconazole were considered pan-resistant. Out of 130 isolates that underwent evaluation of MICs to azoles, 42.3% were found to be resistant to tebuconazole, 18.5% to itraconazole, 20.0% to voriconazole, and 18.5% to posaconazole [28]. The surveillance data showed that despite not being the predominant molecular mechanism responsible for azole resistance, pan-azole resistance was identified in all isolates with the TR34/L98H allele and in all but one isolate with the TR46/Y121F/T289A allele [28]. According to the survey by the Emerging Infections Network (EIN), the azole susceptibility testing of clinical *A. fumigatus* isolates in the US may be underutilized by the providers, and broader surveillance testing might be necessary to aid in the determination of local resistance patterns and further aid in determination of optimal patient management based on the local resistance data [29]. Finally, the most recent survey of 282 clinically significant isolates from North America collected in 2017–2021 as part of the SENTRY Antifungal Surveillance Program detected 11.0% of isolates that were non-wild type to one or more azoles, with 6.0% being non-wild type to isavuconazole, 8.2% to itraconazole, 2.5% to posaconazole, and 1.4% to voriconazole [15].

Prevalence of azole resistance in Asia varies among countries and regions [30]. A prospective study by Dabas et al. evaluated the rate of azole resistance among immunocompromised patients in India [31]. The identification by mycological methods, including direct microscopy and growth on Sabouraud dextrose agar, revealed 32 isolates of *A. fumigatus* among isolated cultures of *Aspergillus* spp. All 32 isolates were tested by the CLSI broth microdilution method and by EUCAST methodology, with the use of ECVs for the CLSI method and EUCAST breakpoints for EUCAST methodology, and a total of 6/706 (0.8%) of azole-resistant *A. fumigatus* isolates were observed among 706 cases of invasive aspergillosis (8 proven and 698 probable) [31]. Among the tested isolates, 18.75% were non-wild type to itraconazole, 3.12% were non-wild type to voriconazole, and 3.12% were non-wild type to posaconazole by both methodologies [31]. Earlier studies from India also showed low rates of resistance [32,33]. The data collected from 12 provinces in China showed the rate of azole resistance in clinical isolates of 2.5% and 1.4% among environmental isolates [34,35,36]. The EUCAST broth microdilution method was utilized for susceptibility testing, and all azole-resistant isolates were identified as *A. fumigatus sensu stricto* [36]. A recent evaluation of 307 clinical isolates collected in 2023-2024 showed 1.2% of azole-resistant *A. fumigatus* isolates [34]. In certain geographical areas, such as the Yunnan province, which is renowned as a prominent agricultural region, exceedingly high rates of resistant isolates have been reported as compared to other regions, with remarkably high rates in greenhouse environments [35,37]. A significantly higher rate of resistance in the environmental surveillance isolates was reported from the Mekong Delta region of Vietnam by Duong et al., which the authors attributed to poorly regulated widespread use of fungicides in agriculture [38]. There is limited data regarding the rates of azole resistance in Africa [39]. Due to voluntary reporting, the data from many countries of Latin America are also limited [40]. Screening of 584 environmental isolates of *A. fumigatus sensu stricto* showed the presence of resistance to at least one azole by EUCAST clinical breakpoints in 6.9% of isolates in Mexico, 8.3% of isolates in Paraguay, and 9.8% of isolates in Peru, with all azole-resistant isolates from Peru demonstrating resistance to all tested azoles (voriconazole, itraconazole, and posaconazole), with all resistant isolates harboring the TR34/L98H *cyp51A* gene mutations [41]. In that study, no azole-resistant isolates were found in Benin, and 2.2% (1/46) of isolates from Nigeria demonstrated low-level resistance to all tested triazole antifungals [41]. In a systematic review by Amona et al., 1.3% of clinical isolates and 17.1% of environmental isolates from the African studies demonstrated resistance to at least one of 4 medical azoles [42]. A significant discrepancy was noted between major geographical regions: low prevalence of azole resistance was observed among the Western African countries, such as Nigeria and Benin, with an average prevalence of azole resistance in that region of 1.6%, whereas a much higher prevalence of azole resistance was observed in the Eastern African countries, such as Tanzania and Kenya, reaching 31%. Such high prevalence may be explained by the extensive use of azole fungicides, mainly 14a-demethylase inhibitors, due to active flower farming in the Eastern African countries [42]. Itraconazole and voriconazole are both used for the treatment of aspergillosis in many African countries [42].

In the retrospective studies of 221 clinical isolates of *A. fumigatus* in Brazil, 1.8% of isolates had high MICs to voriconazole [43]. Among 143 clinical *A. fumigatus sensu stricto* isolates from Lima, Peru, 3 isolates showed resistance to at least one azole with all three exhibiting resistance to itraconazole [44].

Although earlier studies reported that 2% of human isolates (confirmed as *A. fumigatus sensu stricto* by *β-tubulin* gene sequencing) in Australia were non-wild type to one or more azoles (based on CLSI method and using interpretative criteria based on epidemiological cut offs), in the more recent retrospective study of clinically significant isolates from 10 Australasian tertiary centers in 2017–2020, 6.5% *A. fumigatus sensu stricto* isolates were found to be azole resistant [45,46]. Specifically, 2 isolates demonstrated high MICs to voriconazole, posaconazole, and itraconazole, whereas the remaining isolate was susceptible to voriconazole. Susceptibility testing of *A. fumigatus sensu stricto* isolates was performed using a Sensititre^®^ YeastOne™ YO10 panel (TREK Diagnostics, Cleveland, OH, USA) using CLSI breakpoints and ECV cutoff values [46]. Further surveillance in most geographical areas is necessary to better outline the rates of azole resistance.

## 3. Mechanisms of Azole Resistance in *A. fumigatus*

The mutations that confer resistance to azoles can be broadly divided into 2 main categories: mutations of the *cyp51* genes and non-*cyp51* mutations. Mutations can develop during therapy with azoles; however, the environmental selection of resistance species due to exposure to azole fungicides plays a far greater role [30,47]. *A. fumigatus* contains 2 *cyp51* isoenzymes, *cyp51A* and *cyp51B* [48]. Not all *Aspergillus* spp. contain the same number of *cyp51* paralogues; for instance, *A. flavus* and *A. oryzae*, as well as *A. terreus* and *A. carbonarius* carry three paralogues (*cyp51A*, *cyp51B*, and *cyp51C*), whereas *A. fumigatus*, *A. nidulans* and *A. niger* carry two paralogues (*cyp51A* and *cyp51B)* [49]. In *A. fumigatus*, both *cyp51A* and *cyp51B* encode the fungal lanosterol 14α-demethylase, which is responsible for ergosterol synthesis and is the target for the azole antifungals. Inhibition of *cyp51A* and *cyp51B* leads to diversion of ergosterol precursors from the normal biosynthetic pathway and induces accumulation of toxic intermediate products, eventually leading to cell growth arrest and death of the fungal cell [27,50]. Studies show that they act in compensatory fashion, and deletion of the genes encoding one enzyme causes a compensatory increase in the other enzyme [51]. The role of *cyp51A* mutations in the development of azole resistance has been well established in mycological studies [50,52]. The mutations of the *cyp51A* gene associated with the azole-resistance include tandem-repeat associated mutations (TR_34,_ TR_46_, TR_53_, etc.) and point mutations in codons G54, L98, Y121, T289, G138, and M220 [30,53]. The 34-base pair tandem repeat in the promoter region of *cyp51A*, along with a substitution of leucine 98 to histidine (TR_34_/L98H), which usually confers resistance to itraconazole and posaconazole, while voriconazole may retain intermediate susceptibility or develop low-level resistance; however, pan-azole resistance can also develop [28,54,55,56,57]. The presence of both the TR_34_ and L98H is necessary for the development of multi-azole resistance [56]. The other commonly detected mechanism conferring high-level voriconazole resistance and increased MICs to other azoles involves a 46-base pair tandem repeat in the *cyp51A* promoter region accompanied by substitutions of tyrosine 121 for phenylalanine and threonine 289 for alanine (TR_46_/Y121F/T289A) [52,58,59]. Less prevalent tandem-repeat mutations include three and four tandem repeats of the 46-base pair (TR_46_^3^ and TR_46_^4^) [60,61]. The triple 46-base pair tandem repeats (TR_46_^3^) mutation TR_46_^3^/Y121F/M172I/T289A/G448S confers pan-azole resistance and has been described in environmental isolates and samples from airway colonization from 3 patients in the Netherlands [60]. The 53-base tandem repeat (TR_53_) was detected in a patient with *Aspergillus* osteomyelitis in 2006, and the organism exhibited pan-azole resistance [62]. This mutation was also isolated from flower fields in Colombia and recently was reported in the isolates from greenhouses in the southwest of China [35]. The tandem repeat duplications in the *cyp51A* promoter region have been isolated from clinical and environmental isolates, and selective pressure of azole fungicidal agents in the environment has been proposed as the driving mechanism for the development of these mutations [63,64,65,66]. Point mutations in the *cyp51A* gene and non-*cyp51A* mutations, on the other hand, often develop de novo during prolonged treatment with azoles [63]. However, in 2019 Hare et al. reported a case of in vivo development of a novel mechanism of azole resistance, specifically a 120-base pair tandem repeat (TR_120_) associated with prolonged treatment with azole antifungals [67]. A recent study from Brazil reported a novel mechanism of azole resistance associated with TR_46_/F495I recovered from the hospital environment [68]. The isolate harboring the TR_92_ mutation was identified in the flower bulb waste in the Netherlands [69].

Several described point mutations in the *cyp51A* gene, including G54, Y121, G138, P216, F219, M220, A284, Y431, G432, G434, and G448 are responsible for azole resistance [49]. Non-synonymous point mutations at positions G54, M220, and G138 are associated with prolonged treatment with azoles and have been reported to develop in patients receiving treatment for chronic aspergillosis [30,65,70]. The G448S substitution has been identified both from environmental and clinical isolates and has been most frequently reported among patients receiving therapy with voriconazole [71,72].

The role of *cyp51B* mutations in conferring azole resistance remained less clear. The studies have shown that expression of both *cyp51A* and *cyp51B* can be induced by azoles; however, *cyp51B* plays a far lesser role in the development of azole resistance than *cyp51A* [48,50]. The evidence of the presence of *cyp51B* mutations in azole-resistant isolates lacking mutation in the *cyp51A* was provided by Buied et al. [73]. Handelman et al. showed that both overexpression and mutations in *cyp51B* can lead to triazole resistance [50]. However, limited data exist about the clinical implications of the isolates harboring these mutations [27,74].

Although the mechanisms involving alterations in *cyp51A* are responsible for a significant number of azole-resistant isolates, alternative mechanisms of azole resistance have been identified, lacking mutations in the *cyp51A* gene. Some studies estimate that non-*cyp51A* mutations account for about 10% of cases of resistance in environmental strains. However, according to the study by Buied et al., the non-*cyp51A* mutations may be the cause of azole resistance in up to 43% of azole-resistant isolates [64,75]. Among the non-*cyp51A*-mediated mechanisms of resistance, overexpression of drug efflux pumps, alterations in sterol biosynthesis pathways, such as *hmg1* (3-hydroxy-3-methyl-glutaryl-coenzyme A (HMG-CoA) reductase-encoding gene) mutations, exogenous cholesterol import, biofilm formation, alterations in stress response pathways, and certain other modifications have been described [53,70,76,77,78]. Certain regulatory pathways have been described that upregulate the *cyp51A* expression. The single mutation in the *hapE* gene (encoding the *HapE* subunit of the *CCAAT*-binding complex (*CBC*)) occurs outside the *cyp51A* promoter region and its presence increases the *cyp51A* expression [51]. The presence of the mutant *HapE^P88L^* subunit in the multimeric *CCAAT*-binding complex (*CBC*) results in diminished binding of the *CBC* to the *cyp51A* promoter region. The inhibitory effect of the *CBC* on the sterol regulatory element binding protein (*SrbA*) is diminished, resulting in increased *cyp51A* expression and increased MICs to azoles [79,80]. Another factor, the designated *ABC* (ATP-binding cassette) transporter regulator (*ATrR*), plays an essential role in increasing azole resistance by binding to the 34 base-pair region of the *cyp51A* and stimulating its expression [81]. Notably, it also regulates the expression of genes regulating the *ABC*-transporter protein (*AbcG1*), which underscores its role in the development of azole resistance [82]. Loss of the negative cofactor two (*NCT*) complex, a member of a family of transcription regulators, leads to multidrug resistance not only to azoles but also to terbinafine and amphotericin B [83].

Two main superfamilies of efflux pumps facilitating the elimination of azoles from the fungal cell have been described: the *ABC* p (the ATP-binding cassette) and the *MFS* (major facilitator superfamily) [49,77,84]. Not all transporters in these superfamilies act as drug transporters, and the drug transporters within these two superfamilies are referred to as multidrug resistance (*MDR*) or pleiotropic resistance proteins (*PDR*) [49]. *AbcG1* transporter protein plays an important role in the development of azole resistance. The itraconazole-resistant strains exhibited 5-30 times higher basal expression of the *AbcG1* than the resistant strain [85]. Moreover, loss of *AbcG1* from a mutant strain harboring the TR_34_/L98H mutation of the *cyp51A* gene resulted in a significant increase in susceptibility to azoles [86]. Many other drug efflux pumps in the *ABC* superfamily are upregulated during azole exposure (*AfuMDR2*, *abcA-E*, *atrI*, *atrF*, *mdr2-4*); however, upregulation of only certain types of efflux pumps results in increased azole resistance [49,87]. The *MFS*-transporter family is another subfamily of efflux drugs pumps, and their effects on azole resistance are less well studied. Experimental data have shown that deletion of the *mdrA* (member of the *MFS*-transporter family) results in increased susceptibility to itraconazole and voriconazole; however, deletion of *mfs56* had no significant effect on azole susceptibility [84,85].

Upregulation of sterol biosynthesis results in decreased susceptibility to all azole drugs [88]. Mutations in the *hmg1* gene, encoding the HMG-CoA reductase, were found in significant numbers of azole-resistant clinical isolates [88]. Mutations in *hmg1* leading to azole resistance mostly affected the conserved sterol-sensing domain of *hmg1*, possibly leading to impaired negative regulation of Hmg1 activity and accumulation of ergosterol precursors, as well as sustained or increased ergosterol content [88]. However, the study by Hagiwara et al. failed to show a significant effect of ectopic expression of *hmg1* and *erg6* on the development of azole resistance [88,89].

A study published by Wei et al. described two other mechanisms of non-*cyp51A*-mediated azole resistance [90]. Deletion of *algA* (the putative calcium-dependent protein-encoding gene) resulted in increased frequency of azole resistance among the experimental strains, while the mutation in Cox10 (farnesyltransferase) among the *Afcoz10* mutants contributed itraconazole resistance in *algA*-independent manner, likely from decreased intracellular absorption and retention of itraconazole, and could confer cross-resistance to other antifungals [90].

The alterations in stress response mechanisms, such as Ca2+ signaling pathways, play an important role in azole resistance [91]. In *A. fumigatus*, the addition of the Ca2+ chelators ethylene glycol tetra-acetic acid (EGTA) and ethylene diamine tetra-acetic acid (EDTA) results in increased efficacy of both azoles and amphotericin B [91,92,93]. Several other components, including tacrolimus, cyclosporine, calcium channel blockers, and others, exhibit antifungal properties both in combination with antifungals and as a standalone agent [92].

The *A. fumigatus* biofilms are a recognized factor in resistance to azole antifungals [94]. Several mechanisms contribute to the drug-resistance properties of *A. fumigatus* biofilms, including multidrug efflux pumps (i.e., increased transcription of *mdr4*, etc.), production of extracellular matrix (ECM), and, possibly, creation of hypoxic microenvironments. Low-oxygen states may stimulate expression of genes regulating iron and sterol metabolism, possibly contributing to azole resistance under hypoxic conditions [95]. Mismatch repair mechanisms may also play a role in the development of azole resistance [96]. The study by Bottery et al. showed that the G233A variant in the *msh6* gene (part of the mismatch repair system and an essential component of the base–base mispairing recognition system) was significantly associated with the presence of TR_34_/L98H mutation in the *cyp51A* gene, conferring azole resistance among the clade A isolates of *A. fumigatus* [97]. The presence of this *msh6* variant has shown to increase the mutation rate, increasing the chances of gaining spontaneous olorofim resistance [97]. The authors concluded that *msh6* is unlikely to be the cause of azole resistance conferred by the TR_34_/L98H mutation; however, its effect on increasing the mutation rate increases the risk of development of resistance to other antifungals among the azole-resistant strains from this clade [97]. Recently published data also suggest that long noncoding RNAs (lncRNAs) may play an important role in azole resistance in *A. fumigatus* under azole stress [98].

A significant number of other mechanisms of azole resistance in *A. fumigatus* have been described. Overall, our knowledge of genetic and molecular mechanisms of resistance to azole antifungals continues to evolve; however, many questions remain unanswered. Further studies are necessary to further advance our understanding of the mechanisms of azole resistance and their clinical implications.

## 4. Diagnosis of Azole-Resistant Aspergillosis

Traditionally, the detection of azole resistance posed a certain challenge. The first necessary step, recommended by professional societies, is identification of the species of *Aspergillus* [99]. It is recommended to refer the clinically significant *Aspergillus* isolates to the reference laboratory for MIC testing [99]. Testing of up to five different colonies in a clinical specimen for antifungal susceptibility is recommended, as individual colonies may exhibit differing resistance profiles [99]. Clinical breakpoints for interpretation of voriconazole and isavuconazole susceptibility are available for both the European Committee on Antimicrobial Susceptibility Testing (EUCAST) microdilution method and the Clinical & Laboratory Standards Institute (CLSI) method; however, CLSI utilizes epidemiological cut-off values (ECVs) rather than clinical breakpoints for amphotericin B and posaconazole [39,100,101,102,103]. The downsides of culture-based diagnosis of azole-resistant aspergillosis include low yield of fungal cultures and prolonged turnaround time for the susceptibility testing [60]. High-volume culture may have a higher positivity rate compared to conventional cultures and allow for the detection of azole-resistant strains that could otherwise be missed on conventional cultures [104,105].

Molecular methods of azole-resistance diagnosis offer the benefit of non-culture-based detection of common mutations of the *cyp51* gene, associated with azole resistance, which may decrease the turnaround time of the testing [106]. As many cases of IA are diagnosed without positive cultures, the molecular methods offer an additional advantage of detecting resistance-conferring mutations directly from clinical samples without the need for a positive culture [59]. As shown in the study by Lestrade et al., a delay in initiating an appropriate therapy for invasive aspergillosis results in increased mortality among the infected patients, which is why early diagnosis of azole resistance is necessary to facilitate early initiation of appropriate coverage [15]. Reliance on time-consuming culture-based methods may be one of the factors contributing to the delay in appropriate therapy [60].

PCR-based methods are being widely utilized for the diagnosis of invasive aspergillosis, as well as the detection of azole resistance [107,108,109]. Certain limitations in diagnostic accuracy have been previously described, with better performance of *Aspergillus* genus-specific assays compared to species-specific assays [110]. Several commercial molecular testing systems are currently available for diagnosis of azole resistance in invasive aspergillosis. The AsperGenius^®^ assay (PathoNostics, Maastricht, the Netherlands) is a multiplex real-time PCR, consisting of two diagnostic assays. The AsperGenius Species multiplex^®^ offers probes for *A. fumigatus* complex (including *A. fumigatus*, *A. lentulus*, *A. udagawae*, and *A. viridinutans*) and a probe for *Aspergillus* spp. (*A. fumigatus complex*, *A. terreus*, *A. flavus*, and *A. niger*), while the AsperGenius Resistance multiplex^®^ assay aids in detection of *cyp51A* gene mutations in *A. fumigatus* associated with resistance (TR34, L98H, Y121F, and T289A) [106,111,112]. An important diagnostic feature of the assay is its ability to differentiate wild-type strains from mutant strains in cases of mixed infections [3,112,113]. It is validated for use on BAL, serum/plasma, but not validated for biopsies or CSF samples [112,113,114]. Several clinical studies have suggested potential benefit of the AsperGenius^®^ assay in clinical practice [111,115,116]. The assay can be beneficial for both clinical practice and epidemiological surveillance, especially when combined with culture-based methods [117]. It may offer an additional advantage of more rapid diagnosis as compared to in-house PCR assays with subsequent DNA sequence analysis [118].

The MycoGenie^®^ *Aspergillus fumigatus* and resistance assay (Pessac, France) detects the presence of *A. fumigatus* in the sample as well as the presence of the TR_34_/L98H resistance-associated mutation in biopsies, serum, and respiratory tract samples [109]. No cross-reactivity was detected with *A. flavus*, *A. niger*, *A. nidulans*, *A. versicolor*, *A. terreus*, as well as other tested organisms [119]. In the study by Mikulska et al., the resistance was detected in one patient which resulted in a change in therapy and clinical improvement [120]. The Fungiplex^®^ *Aspergillus* Azole-R IVD PCR (Bruker Daltonik GmbH, Bremen, Germany) is a real-time PCR kit validated for detection of TR_34_/L98H and TR_46_/Y121F/T289A mutations from DNA extracted from serum, plasma, and BAL samples [109]. The Fungiplex^®^ *Aspergillus* Azole-R IVD PCR is not available in the US.

Pyrosequencing offers the advantage of screening for all *cyp51A* resistance-conferring polymorphisms, simultaneously detecting both wild-type and resistant alleles; however, it is not yet widely available [121]. MALDI-TOF holds promise as a potential tool for detection of azole-resistant *A. fumigatus* [122,123,124]. Whole-genome sequencing (WGS) has been utilized on clinical and environmental specimens to investigate the routes of acquisition of azole-resistant strains, as well as the evaluation of differences in virulence potential between clinical and environmental strains [125,126]. Utilization of WGS is currently mostly limited to certain specific purposes [47,127]. A next-generation sequencing-based clinical assay to predict the phenotypic susceptibility of *A. fumigatus* to azoles and identification of wild-type isolates has been developed, but further data are required to establish its utility in clinical practice [128].

## 5. Azole-Resistant A. fumigatus: Impact on Treatment Strategies

The initial choice of therapy should be guided by the local prevalence of azole resistance [129,130]. It was suggested by the expert panel that voriconazole can be utilized as initial therapy if the prevalence of azole resistance is less than 5% [99,129]. In case of high level of azole resistance (over 10% as defined by a panel of experts), empiric therapy of voriconazole plus echinocandin or liposomal amphotericin B was proposed as the initial treatment of choice [129]. Combination therapy is now the recommended initial treatment in the Netherlands [131]. Subsequent adjustments of the initial antifungal therapy are based on several factors, including the results of susceptibility testing [129]. Suggestions on the choice of empiric antifungal regimen for invasive pulmonary aspergillosis based on local rates of azole resistance are outlined in Table 1.

The IDSA guidance advises against routine antifungal susceptibility testing unless there is a suspicion of an azole-resistant *A. fumigatus* being the causative agent of the disease, a lack of response to appropriate treatment with an azole antifungal, or for epidemiological purposes [23]. However, according to the 2017 ESCMID-ECMM-ERS guideline, antifungal susceptibility testing should be performed in clinically relevant isolates of *A. fumigatus* in cases of invasive disease, except in azole-naïve patients with low resistance rates based on surveillance programs [99]. At the very minimum, testing for susceptibility to voriconazole and itraconazole is required. Posaconazole resistance without itraconazole resistance has not been reported in the literature [99]. If the isolate demonstrates a voriconazole MIC of 2 mg/mL, a combination of voriconazole and an echinocandin or liposomal amphotericin B can be utilized [99]. For isolates with voriconazole MIC>2 mg/mL, liposomal amphotericin B is suggested as first-line therapy [99,129,130]. Voriconazole+ anidulafungin, posaconazole + caspofungin, amphotericin B lipid complex, and monotherapy with an echinocandin (caspofungin or micafungin) are suggested as alternative options in documented azole resistance [99,132]. A switch to a different class is recommended [99].

Use of voriconazole as monotherapy in experimental models of voriconazole-resistant aspergillosis showed that higher voriconazole exposures are needed for isolates with higher MICs and will be associated with higher risks of toxicity [133]. Monotherapy with azoles in azole-resistant *A. fumigatus* is generally not recommended [129]. Some studies showed synergistic effects of an anidulafungin/voriconazole combination in voriconazole-susceptible isolates in vivo; however, among animal models infected with voriconazole-resistant isolates, additivity, rather than synergy, was observed with reduced effect in voriconazole-resistant cases [134]. However, the study by Krishnan-Natesan et al. demonstrated in vitro synergy of a voriconazole/anidulafungin combination against both voriconazole-susceptible and -resistant isolates [135]. Another in vitro study utilizing a voriconazole/anidulafungin combination also showed that although an additive effect between voriconazole and anidulafungin was apparent—a comparable antifungal effect in voriconazole-resistant isolates could only be achieved with proportionally greater exposures to voriconazole [136]. Another study of the effects of voriconazole/anidulafungin combination in *A. fumigatus* with different in vitro susceptibilities to voriconazole in vitro showed dose-dependent effects, with synergistic effects at low drug exposures and antagonistic effects at higher drug exposures [137].

The susceptibility to isavuconazole, as shown in several studies, closely parallels that of voriconazole among both voriconazole-susceptible and resistant strains [56,138]. Experimental data suggest that high-dose isavuconazole may be beneficial in cases of *A. fumigatus* with an MIC of 2 mg/L, although it will also require close monitoring due to potential drug–drug interaction and toxicity [56]. The combination of isavuconazole and anidulafungin in vitro showed synergy against a significant number of wild-type and azole-resistant isolates; however, it failed to demonstrate benefit against isolates harboring high-level resistance against isavuconazole, such the TR46/Y121F/T289A mutation [139]. The 2017 ESCMID-ECMM-ERS guideline does not endorse monotherapy with posaconazole as a treatment option; however, the use of a posaconazole+caspofungin combination is suggested as a salvage option [99]. High-dose posaconazole with serum trough level >3 mg/L was investigated as an option for invasive mold infections, including azole-resistant aspergillosis, in the study by Schauwvlieghe et al. [140]. The authors concluded that high-dose posaconazole might be considered as a therapeutic option, although close monitoring for the development of adverse events and exposure would be necessary [140]. Recent experimental data suggested a possible therapeutic benefit of the combination of posaconazole with tacrolimus against both azole-sensitive and azole-resistant strains [141].

Although susceptibility to echinocandins is not affected by the presence of azole resistance and caspofungin has activity against *A. fumigatus*, the success rate of treatment is low [129,133,142,143,144].

Novel antifungals with new mechanisms of action may become important tools in the management of infections caused by azole-resistant *A. fumigatus* [145,146]. Ibrexafungerp, a member of the triterpenoid class, acts as an inhibitor of synthesis of beta-D-glucan in the fungal cell wall, exhibiting fungistatic activity against *Aspergillus* species [146,147,148]. In vitro data suggest that ibrexafungerp is active against both azole-susceptible and azole-resistant strains of *A. fumigatus*, as well as cryptic species [147,149]. The combinations of ibrexafungerp+an azole (voriconazole or isavuconazole) or amphotericin B showed in vitro synergistic activity against all tested wild-types strains; however, the effects on azole-resistant strains were less impressive in one study [150]. The phase 3 FURI trial (NCT03059992) assessing the use of ibrexafungerp for invasive candidiasis and chronic or invasive aspergillosis, including azole-resistant strains, with intolerance or documented failure of currently approved drugs has been recently completed with some of the results available for review; however, full data on efficacy in azole-resistant *A. fumigatus* in this study are not yet available [151].

Olorofim reversibly inhibits fungal dihydroorotate dehydrogenase, which results in disruption in pyrimidine synthesis, DNA/RNA synthesis, and other essential cellular processes in the fungal cell [152,153,154,155]. It showed activity against azole- and amphotericin B-resistant *Aspergillus* species, including cryptic species [152,153,154]. Prolonged exposure to olorofim was shown to have fungicidal activity, resulting in hyphal swelling and lysis of the fungal cells [156]. In vitro cross-resistance with other medical antifungal agents has not been widely reported; however, recently published data suggest the development of cross-resistance between olorofim and the agrochemical fungicide ipflufenoquin [157,158]. However, the in vitro data, published by van Rhijn et al., suggest that the azoles may induce the upregulation of the pyrimidine biosynthesis pathway, which results in antagonism between olorofim and azoles [159]. Olorofim showed antibiofilm properties during the early stage of biofilm formation, while the mature biofilm was resistant to treatment with olorofim [160]. The results of the open-label, single-arm Phase II b study of olorofim for the management of invasive fungal diseases with limited treatment options were recently published [157]. In the modified intention to treat (mITT) group, 101 patients with invasive aspergillosis were included, with 23 (23%) with known or predicated resistance to all licensed agents. The primary outcome of the study was global response rate at day 42, assessed based on a composite of clinical, radiological, and mycological responses. The outcome was defined as success if a complete or partial improvement occurred in all three components, whereas the definition of failure included stable disease or progression on any component or death from any cause. Successful global response at day 42 was noted among 35 (34.7%) patients and among 34 (33.7%) patients at day 84. However, when the stable disease was included in the definition of successful global response, the successful global response among the patients with invasive aspergillosis was observed in 65 (64.4%) patients at day 42 and in 55 (54.5%) patients at day 84 [157]. The phase III study comparing the efficacy, safety, and tolerability of olorofim to Ambisome followed by standard of care in patients with invasive aspergillosis, including cases refractory to azole therapy (OASIS, NCT05101187), is currently in the recruitment phase.

Fosmanogepix is a prodrug of manogepix, first in its class [161,162,163]. It inhibits glycosylphosphatidylinositol-anchored wall transfer protein-1 (Gwt1), which leads to impaired maturation of cell wall mannoproteins, essential for cell wall integrity. This results in the inability of the fungal cells to adhere to mucosal/epithelial surfaces, eliminating the initial steps of the infectious process [161,164]. The recently published results of the AEGIS trial demonstrated an acceptable safety profile among patients with limited treatment options [163]. The combination of fosmanogepix with Liposomal Amphotericin B resulted in improved median and overall survival, as well as an increase in resolution of an invasive mold infection and decrease in fungal burden in murine models [165].

Rezafungin, a long-acting echinocandin, has a long half-life (~80 h after the first dose and ~150 hrs after the second dose) and a good safety profile with mostly mild adverse events and a lack of clinically significant drug–drug interactions [166,167]. In vitro data are suggestive of potent activity against azole-resistant *A. fumigatus*, as well as cryptic *Aspergillus* species [168]. Extended-interval infusions of rezafungin showed improved survival in a neutropenic murine model of disseminated aspergillosis [169]. It may become an important tool in the management of azole-resistant aspergillosis as a component of a combination therapy, although further clinical data are needed [145].

The novel azole, opelconazole, was designed in inhalation form to decrease the systemic azole toxicity [145,170]. It showed synergistic activity in vitro with systemic azoles against azole-susceptible and azole-resistant *A. fumigatus* in a human lungs model and in the neutropenic murine model [171]. Opelconazole has also demonstrated extensive activity against both azole-susceptible and azole-resistant *A. fumigatus* [172]. Further studies to establish its clinical role as part of combination therapy in azole-resistant *Aspergillus* infections are required. Table 2 briefly summarizes the data on the potential utility of novel antifungals in azole-resistant invasive aspergillosis.

## 6. Conclusions

Due to advances in medical research and better outcomes and survival of recipients of stem cell and solid organ transplants, as well as increased survival rate of patients with other predisposing conditions, we can anticipate an increase in populations at risk for infections due to azole-resistant *A. fumigatus*. Extensive use of azole fungicidal agents, as well as increased utilization of azole prophylaxis and treatment, may further promote development of resistance to medical azoles. Delays in initiation of appropriate therapy, challenges in rapid detection of azole resistance, lack of epidemiological data, and limited therapeutic options contribute to high mortality rates in azole-resistant aspergillosis. Extensive surveillance with expansion to areas with currently limited surveillance data would help determine the prevalence of resistance and inform empiric therapeutic approach. More studies are also necessary to evaluate the efficacy of novel therapeutics against azole-resistant *A. fumigatus* and determine their role in management of azole-resistant aspergillosis.

## Figures and Tables

**Table 1:** Suggested choices of primary empiric antifungal regimen for invasive pulmonary aspergillosis caused by *A. fumigatus* based on local rates of azole resistance [23,99,129].

Rate of Azole-Resistance	Azole-Resistance < 5%	Azole-Resistance 5–10%	Azole-Resistance > 10%
Suggested initial choice of antifungal therapy	Treatment choices guided by the national or international therapy guidelines (IDSA, ESCMID-ECMM-ERS, etc.) [129]: Isavuconazole [23,99], Voriconazole [23,99,129], Liposomal Amphotericin B [23,99] Other options depending on specific populations: Voriconazole +Anidulafungin or Caspofungin [99], Caspofungin [99], Itraconazole [99], Amphotericin B Lipid Complex [99], Micafungin [99]	Voriconazole monotherapy [129], Voriconazole+ Echinocandin [129], Liposomal Amphotericin B [129]	Voriconazole+ Echinocandin [129], Liposomal Amphotericin B [129]

**Table 2:** Novel antifungals and their potential role in management of azole-resistant aspergillosis [145,146,147,149,151,152,154,157,160,161,162,163,164,165,172,173,174,175,176,177].

Drug	Class	Mechanism of Action	Future Clinical Applications	Comments on Potential Role in Azole-Resistant Aspergillosis
Ibrexafungerp	Triterpenoid	Inhibits Beta-D-glucan synthesis and impairs fungal cell wall integrity	Invasive candidiasis including *C.auris* and *C.glabrata*, invasive aspergillosis including resistant or with limited treatment options, invasive fungal infections refractory or intolerant to standard of care Treatment of vulvovaginal candidiasis (VVC). Reduction in incidence of recurrent VVC (RVVC).	In vitro activity against azole-resistant species [147,149] FURI (NCT03059992) trial: evaluation of safety and efficacy in cases refractory or intolerant to standard therapy: recently completed, however data on efficacy in azole-resistant *A. fumigatus* in this study are not yet available [151]. Consideration as a part of combination therapy in azole-resistant aspergillosis (i.e., with azoles or Liposomal Amphotericin B) [145].
Olorofim	Dihydroorotate dehydrogenase inhibitor	Reversibly inhibits fungal dihydroorotate dehydrogenase, resulting in disruption in pyrimidine synthesis	Treatment of invasive mold infections, including refractory or resistant aspergillosis, infections due to *Lomentospora prolificans*, *Scedosporium*, and *Scopulariopsis* species, coccidioidomycosis, invasive fusariosis, endemic mycoses, refractory to standard of care or with limited treatment options, refractory CNS coccidioidomycosis or with limited treatment options.	In vitro and in vivo activity against azole-resistant strains [152,154,160,174,175,176,177] FORMULA-OLS trial(NCT03583164): olorofim as treatment for invasive fungal infection with few or no therapeutic options: recently published [157]. OASIS trial (NCT05101187): olorofim vs. liposomal amphotericin B followed by standard of care in case of limited treatment options: recruitment phase. Possible use as monotherapy or in combination [145].
Fosmanogepix	Gwt1 inhibitor	Impaired maturation of cell wall mannoproteins results in loss of cell wall integrity, impaired adhesion, pathogenicity, and evasion of host recognition [164]	Invasive candidiasis (including *C.auris*, however excluding *C.krusei*), aspergillosis, scedosporiosis, fusariosis, mucormycosis, cryptococcosis, and coccidioidomycosis, endemic mycoses.	Activity against azole-resistant strains and favorable safety profile (AEGIS trial) [161,162,163,174]. Possible use as monotherapy or in combination, i.e., synergistic activity with Liposomal Amphotericin B [145,165].
Opelconazole	Triazole	Inhibitor of lanosterol 14-demethylase	Invasive pulmonary aspergillosis, allergic bronchopulmonary aspergillosis, chronic pulmonary aspergillosis, COVID-19 associated pulmonary aspergillosis.	Activity against azole-resistant *A. fumigatus* [171,172] Consideration as part of combination therapy in azole-resistant aspergillosis [145] OPERA-T (NCT05238116); systemic antifungal +opelconazole vs. placebo in refractory invasive aspergillosis: ongoing.
Rezafungin	Echinocandin	Inhibition of the Beta-D-glucan synthase enzyme complex	Candidemia/invasive candidiasis in adults with limited or no alternative treatment options, treatment of infections caused by *Aspergillus* spp. and *Pneumocystis jirovecii*. Prophylaxis of invasive fungal diseases in recipients of allo-HSCT.	Demonstrated in vivo and in vitro activity against azole-resistant *A. fumigatus* [145,168,171,177]. Consideration as a part of combination therapy with another mold-active agent [145]

## Data Availability

No new data were created or analyzed in this study. Data sharing is not applicable to this article.
